# Brain stimulation in attention deficits after traumatic brain injury: a literature review and feasibility study

**DOI:** 10.1186/s40814-021-00859-3

**Published:** 2021-05-31

**Authors:** Ève Boissonnault, Johanne Higgins, Geneviève LaGarde, Dorothy Barthélemy, Céline Lamarre, Jehane H. Dagher

**Affiliations:** 1 Physical Medicine and Rehabilitation Service, Université de Montréal, Montreal, QC Canada; 2 Institut universitaire sur la réadaptation en déficience physique de Montréal (IURDPM), 6300, avenue de Darlington (Pavillon Gingras), Montréal, QC H3S 2J4 Canada; 3 Centre for Interdisciplinary Research in Rehabilitation of Greater Montreal (CRIR), Montreal, QC Canada; 4 School of Rehabilitation, Faculty of Medicine, Université de Montréal, Montreal, QC Canada

**Keywords:** Attention, Brain injuries, Traumatic, Rehabilitation, Transcranial direct current stimulation, Transcranial magnetic stimulation

## Abstract

**Background:**

After a traumatic brain injury, disturbances in the attentional processes have a direct negative effect on functional recovery and on return to complex activities. To date, there is no good attention remediation treatment available. The primary objective of this review and pilot study is to provide an overview of the research evidence and to evaluate the feasibility of implementing a tDCS protocol to improve attention disorders in patients with mild complicated to severe subacute TBI, hospitalized in an inpatient rehabilitation facility. Our secondary objective is to extract preliminary data and observational information on participants’ response to treatment.

**Methods:**

Participants were recruited from a consecutive series of patients admitted to the TBI unit of a subspecialized regional rehabilitation center. They received a 20-min tDCS stimulation 3 times a week for 3 weeks. A neuropsychological evaluation was performed before and after the intervention. We collected participants’ sociodemographic and clinical characteristics as well as information about satisfaction, tolerability, and adverse effects.

**Results:**

One hundred sixty-four patients were admitted between September 2018 and January 2020. One hundred fifty-eight were excluded, and 6 patients with presumed attentional deficits were enrolled. None completed the protocol as intended. No major side effects occurred.

**Conclusion:**

Non-invasive brain neurostimulation is promising to enhance attention deficits in patients with TBI. Implementation of a tDCS protocol to fulfill this purpose in an intensive inpatient rehabilitation center has its limitations. We made recommendations to facilitate the implementation of similar projects in the future.

**Trial registration:**

ISRCTN, ISRCTN55243064. Registered 14 October 2020—retrospectively registered.

## Key messages regarding feasibility

- NIBS is promising for the treatment of attention deficits in persons with TBI undergoing inpatient rehabilitation.
- Complexity of organizing tDCS sessions within inpatient rehabilitation schedule needs careful consideration.
- Recommendations include the assignment of a dedicated research coordinator, completion of the study in an outpatient setting, and a maximum of 2 NIBS sessions per week.

## Background

In the USA, annually, over 20,000 adults receive inpatient rehabilitation for moderate-to-severe traumatic brain injury (TBI) [1]. Studies show that only 26.1% of individuals with a moderate-to-severe TBI reach a disability-free global level of functioning at 5 years post-injury [1]. With regard to cognitive function, 49.1% of patients with TBI are not fully independent after 5 years [1].

In the acute stage of TBI, the primary injury refers to lesions related to the trauma mechanism [2–4]. Among focal insults, contusion is the commonest and most often located in the frontal and temporal lobes where the fragile brain tissue comes in contact with irregular bony protuberances of the anterior and middle cranial fossa [2, 4, 5]. The secondary injury occurs up to several weeks after the primary injury and can result from excitotoxicity, cerebral edema, ischemia, and neuroinflammation [4, 6]. Then, functional cell plasticity and remyelination prevail within the first 3 months after insult. Therefore, we can expect the greatest recovery in the subacute phase [4, 6]. In the acute stage of TBI, impaired consciousness and post-traumatic amnesia are mostly seen, whereas attentional deficits, memory impairments, communication disorders, altered processing speed, and executive dysfunction are noted in the subacute period [5].

Attention is a complex mental activity that refers to how individuals receive and process internal and external stimuli [7]. Sustained attention is the capacity to maintain a constant focus on a continuous and repetitive activity, divided attention is the ability to pay attention and to process information coming from two or more sources, and selective attention is the faculty to maintain focus on one trigger or idea for a short moment without being distracted by environmental or competitive stimuli [8, 9]. Those disturbances in the attentional processes have a direct negative effect on functional recovery and on return to complex activities after TBI [5]. Thus, attentional impairments represent an important target in the rehabilitation intervention in subacute phases of TBI. Traditional attention remediation programs consisting of a battery of pen and paper tasks, informatics software, and coping strategies have shown their usefulness, but the evidences supporting specific intervention strategies remains limited [10].

### Transcranial neurostimulation

To influence neuronal plasticity, non-invasive brain neurostimulation (NIBS) uses an extrinsic-induced electrical stimulus to modulate neuronal excitability, synaptic strength, and dendritic connections. Long-term potentiation (LTP) increases synaptic transmission between two neurons by modulating gamma-aminobutyric acid (GABA)ergic and glutamatergic synapses, while long-term depression (LTD) decreases it and reduces glutamate excitotoxicity and GABA-mediated inhibition [4, 11, 12]. The two most studied modalities are transcranial direct current stimulation (tDCS) and repetitive transcranial magnetic stimulation (rTMS). Transcranial direct current stimulation allows a weak direct current to flow from the anode to the cathode placed on the scalp to modify the resting membrane potential and modulate the activity level of spontaneous excitatory neurons. Anodal tDCS increases the excitability of the cortex, and cathodal stimulation decreases it. rTMS generates a magnetic field that induces an electric current to neural tissue. High-frequency stimulation facilitates neuronal efficiency, while low-frequency rTMS reduces it [3, 12]. Depending on the frequency of repetitive transcranial magnetic stimulation (rTMS) or the polarity of the tDCS, these techniques can induce LTP-like or LTD-like effects that last beyond the stimulation time frame [3, 4, 11, 12].

For this research project, tDCS was determined a better option than rTMS. First, its convenience such as portability allows a better adherence to treatment. Furthermore, the current produced by tDCS being less focal than the rTMS is of interest given the diffuse nature of the TBI. Lastly, tDCS has not been reported to induce seizures and thus appears to be a safer choice [3, 13, 14].

### Neurostimulation and cognitive impairment

To date, studies have mainly explored the use of neurostimulation to improve psychiatric disorders, stroke rehabilitation, and healthy subjects [3, 12, 13]. Among those, Dubreuil-Vall et al. concluded that stimulating the left dorsolateral prefrontal cortex (DLPFC) leads to a significant improvement in reaction time [15], and Jones et al. demonstrated a significant improvement on the working memory task with stimulation of the right frontoparietal area [16].

Research on patients with TBI has been pursued only recently because NIBS and especially rTMS are regarded as relative contraindications given that TBI increases neuronal excitability and seizure risk [13]. Post-traumatic epilepsy has an incidence of about 5% in patients with closed head injuries and 50% in those with a penetrating injury [3]. Seizures are the most concerning adverse events in the therapeutic application of NIBS. If compensatory safety steps are taken, experts agree that the expected benefit justifies the increased risk [3, 13]. In fact, literature reports only two incidences of seizures in patients with TBI, both in studies using rTMS and none in studies using tDCS [13].

Two systematic reviews highlight the use of NIBS for improvement of motor function, memory, tinnitus, alteration of consciousness, and depression [11, 13]. The most recent concluded that tDCS is a safe and non-invasive neuromodulatory technique that may be best combined with other therapeutics to improve cognitive and motor outcomes [11]. Out of the 14 studies reviewed, seven used tDCS to improve responsiveness in patients with disorders of consciousness, six in cognition and one in motor outcomes. They underline the challenge in conducting clinical trials due to the heterogeneous rehabilitation interventions and the difficulty in targeting a cognitive function as it does not correspond to a delineated network as compared to motor function [11].

Only eight papers were published on the impact of NIBS on attention after TBI with abnormal structural imaging in human subjects [7, 17–23]. Four research teams used rTMS [17–19, 23], and four used tDCS [7, 20–22]. Five of the protocols targeted the DLPFC [7, 20–23], four of which chose the left side [7, 21–23]. Because of the studies’ low statistical power, wide variability in type of attention evaluated, study design, and lack of consistency between attention type and the tests chosen to assess it, it was difficult to draw a conclusion on NIBS’ effectiveness. Nonetheless, the majority of results were promising and exhibit a positive trend, except for one pilot study [21] and one randomized double-blind trial [23] which failed to show significant differences between the groups. Four studies showed an improvement in some form of attention [17–20], two studies showed an improvement in reaction time [7, 20], one study reported gains in activities of daily living [18], and three studies revealed favorable visible changes in brain imaging (magnetic resonance imaging (MRI), functional MRI, photon emission computed tomography (SPECT) scans) [17, 18, 20]. Ulam et al.’s study is the only one with all 26 patients studied in the subacute phase (the first 6 months following trauma). It showed significant electroencephalogram (EEG) changes after 10 consecutive sessions of tDCS compared to the sham group, suggesting improved regulation of cortical excitability that correlates with improved performance on neuropsychological tests [22]. A study by Kang et al., which also included subacute patients with TBI, demonstrated that a single session of anodal tDCS applied to the left dorsolateral prefrontal cortex (DLPFC) improved attention compared to the sham stimulation, suggesting its potential role in improving attention [7]. This finding is consistent with literature reporting the crucial role that DLPFC plays on attention [5, 20, 22].

In accordance with theory and previous studies, we hypothesized that anodal tDCS applied on the left DLPFC has the potential to enhance attention in patients with mild complicated to severe TBI. Although TBI causes multiple cognitive impairments, we targeted specifically the attentional deficit because of its impact on rehabilitation.

The primary objective of this pilot study was to evaluate the feasibility of implementing an intervention protocol consisting of nine sessions of tDCS to improve attention disorders in patients with mild complicated to severe subacute TBI hospitalized in a rehabilitation facility. Our secondary objective was to extract preliminary data and observational information on participant responses to treatment on selective attention.

## Methods

### Participants

Participants were recruited from a consecutive series of patients admitted to a functional rehabilitation TBI unit at the Institut universitaire sur la réadaptation en déficience physique de Montréal (IURDPM), a subspecialized regional rehabilitation center in Montreal, Canada. Eligible patients were approached by a physician or nurse external to the research team, and written informed consent was obtained from all participants. We arbitrarily agreed that five subjects would be sufficient to test the feasibility of our protocol.

Inclusion criteria were (1) current hospitalization; (2) age 18 years old or more; (3) diagnosis of mild complicated, moderate, or severe TBI [24]; (4) attentional impairment as per qualitative clinical assessment; (5) French or English speaking; (6) tolerance to 45 to 60 min neuropsychology evaluation; and (7) capacity to consent in accordance with the Nova Scotia Hospitals Act [25].

Exclusion criteria were (1) history of neurological disease not resulting from the current TBI (e.g., stroke, multiple sclerosis, neurodegenerative disorders), (2) psychiatric illness (e.g., depression, schizophrenia, anxiety disorders), (3) aphasia and compromises in understanding instructions, (4) significant deafness or blindness, (5) contraindication to tDCS (e.g., seizure, extensive cranial vault lesion, pregnancy or breastfeeding, pacemaker, cochlear implants, or cerebral metal implanted device or clip), (6) scar or skull deformity at the site of electrode placement, (7) epileptogenic medication, and (8) penetrating TBI.

### Study design

This study was designed for patients to receive stimulation over a period of 4 weeks, as presented in Table 1. Sociodemographic information was collected on patients’ sex, age, ethnicity, and education from patients’ interviews and medical records.

**Table 1:** Study design

T0	T1	T2	T3	T4	T5	T6	T7	T8	T9
1 week or less	3×/week × 3 for a total of 9 tDCS active stimulation sessions								
Sociodemographic questionnaire, TEA (EC, ECD), Ruff 2&7, CPT-3, D-KEFS (Stroop), WAIS-IV (DS, CD, BD)	First stimulation session	Adverse effects questionnaire							Satisfaction and tolerability questionnaire, TEA (EC, ECD), Ruff 2&7, CPT-3, D-KEFS (Stroop), WAIS-IV (DS, CD, BD)

*Abbreviations*: *TEA* Test of Everyday Attention, *EC* elevator counting, *ECD* elevator counting with distraction, *CPT-3* Conners’ Continuous Performance Test 3rd Edition, *D-KEFS* Delis-Kaplan Executive Function System, *WAIS-IV* Wechsler Adult Intelligence Scale–fourth edition, *DS* Digit Span, *CD* Coding, *BD* Block Design

All subjects underwent a neuropsychological evaluation (T0). Subjects were intended to start the tDCS protocol, which consisted of 20-min tDCS active stimulation 3 times a week for 3 weeks for a total of 9 sessions (T1-T9). Customized questionnaires to evaluate satisfaction (Table 3), tolerability, and adverse effects (Table 4) as well as a post-treatment neuropsychological evaluation were administered after the last session (T9). In between stimulation sessions, participants pursued their regular functional intensive rehabilitation.

### Transcranial direct current stimulation

The tDCS was applied by experienced researchers. Skin at the site of the electrodes was cleansed with alcohol. Two saline-soaked electrodes of 25cm^2^ each (5 × 5 cm) were placed on the scalp: an anode (excitatory electrode) overlying the left DLPFC, and a cathode (reference electrode) above the right supraorbital area (respectively location F5 and F2p, according to the 10–10 electroencephalography system for electrode placement) [26]. The electrodes were inserted into 5 × 5-cm sponges soaked in saline and fixed with two elastic bands, and tDCS was delivered using a battery-driven tDCS stimulator (Model 1300A; Soterix Medical, New York, NY). Current delivery was monitored throughout the testing. Patients underwent 20-min sessions of tDCS at an intensity of 2 mA and a current density of 0.08 mA/cm^2^. Those parameters were considered safe [14]. Subjects, seated in a quiet room during stimulation, were asked to remain inactive. Participants’ tolerance to the stimulation, satisfaction, tolerability, and adverse effects were recorded at the end of the procedure.

### Outcome measures

#### Primary outcome

To assess the protocol’s feasibility, we collected data on recruitment, refusal, retention, and therapeutic compliance rate. We registered the number of, and reasons for, withdrawals. To monitor tolerance, adverse effects, and safety, each subject was invited to fill out a customized questionnaire (Table 4) adapted from those provided by Brunoni et al. [14].

#### Secondary outcome

We hypothesized that selective attention should improve with tDCS. Specific tests were administered before and after stimulation. The evaluation is routinely given by IURDPM’s neuropsychologists and takes between 45 and 60 min in total to complete. All tests were administered by an experienced neuropsychologist. All tests used have a good test-retest reliability and validity for subjects with TBI [9, 27–30].

Main measures used were: the *Test of Everyday Attention *(*TEA*), sensitive to test selective attention [9]; the *Ruff 2 &7 Selective Attention Test*, a measure of sustained and selective attention [8]; *the Conners’ Continuous Performance Test 3rd Edition* (CPT-3), for visual sustained attention, vigilance, impulsivity, and inattentiveness [31]. Secondary measures related to attention used were the *Stroop* from the *Delis-Kaplan Executive Function System* (D-KEFS) for selective attention [32] and the *Digit Span* and *Coding* subtests from the *Wechsler Adult Intelligence Scale–fourth edition* (WAIS-IV) respectively assess working memory and processing speed [33, 34]. Secondary measures not related to attention were the *Block Design* subtest from the WAIS-IV that evaluates the ability to analyze and synthesize abstract visual stimuli. The results were used to measure the specificity of our stimulation intervention. It is therefore not expected to improve compared with measures related to attention [33, 34].

## Results

**Fig. 1:**
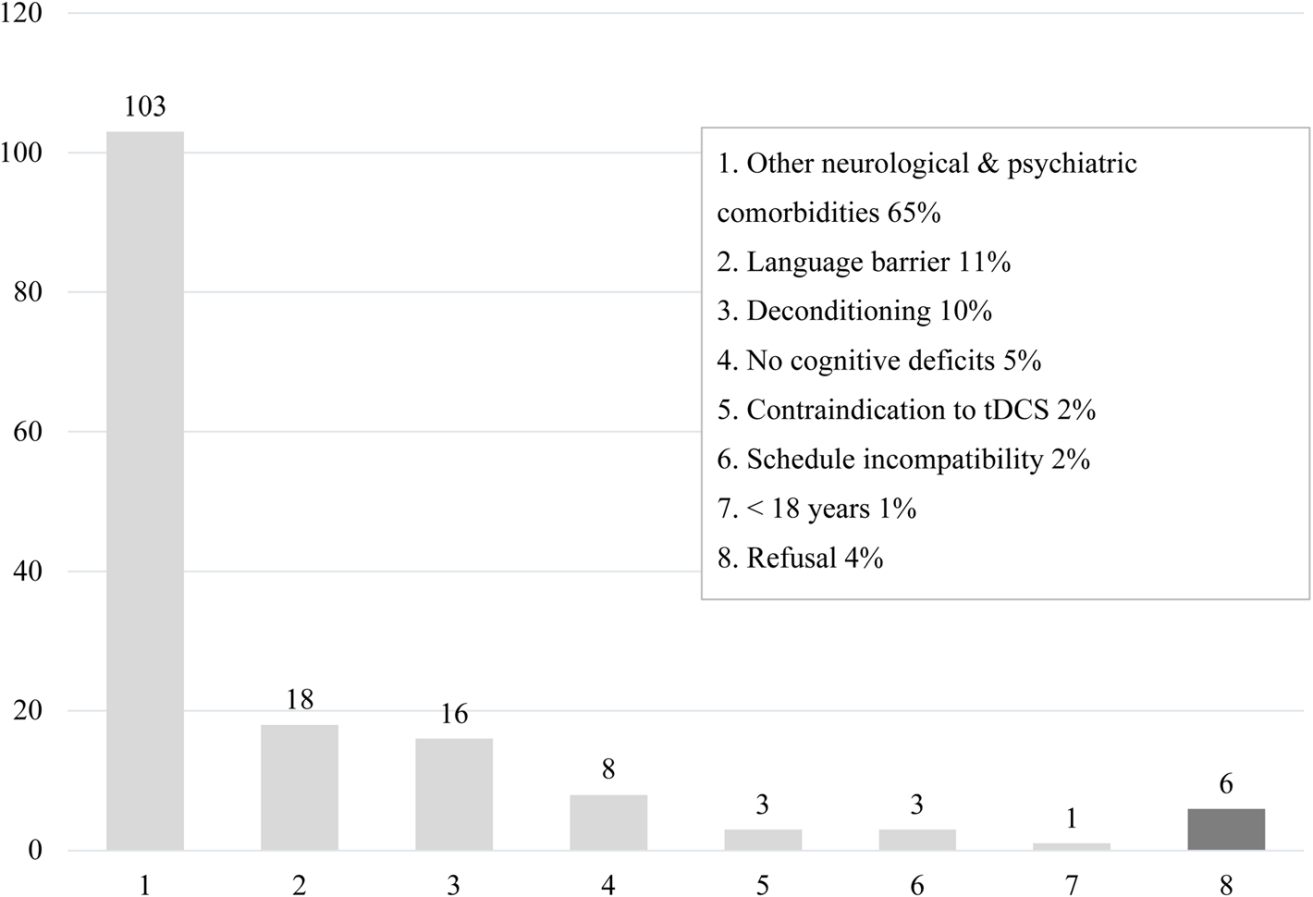
Exclusions and refusals

**Fig. 2:**
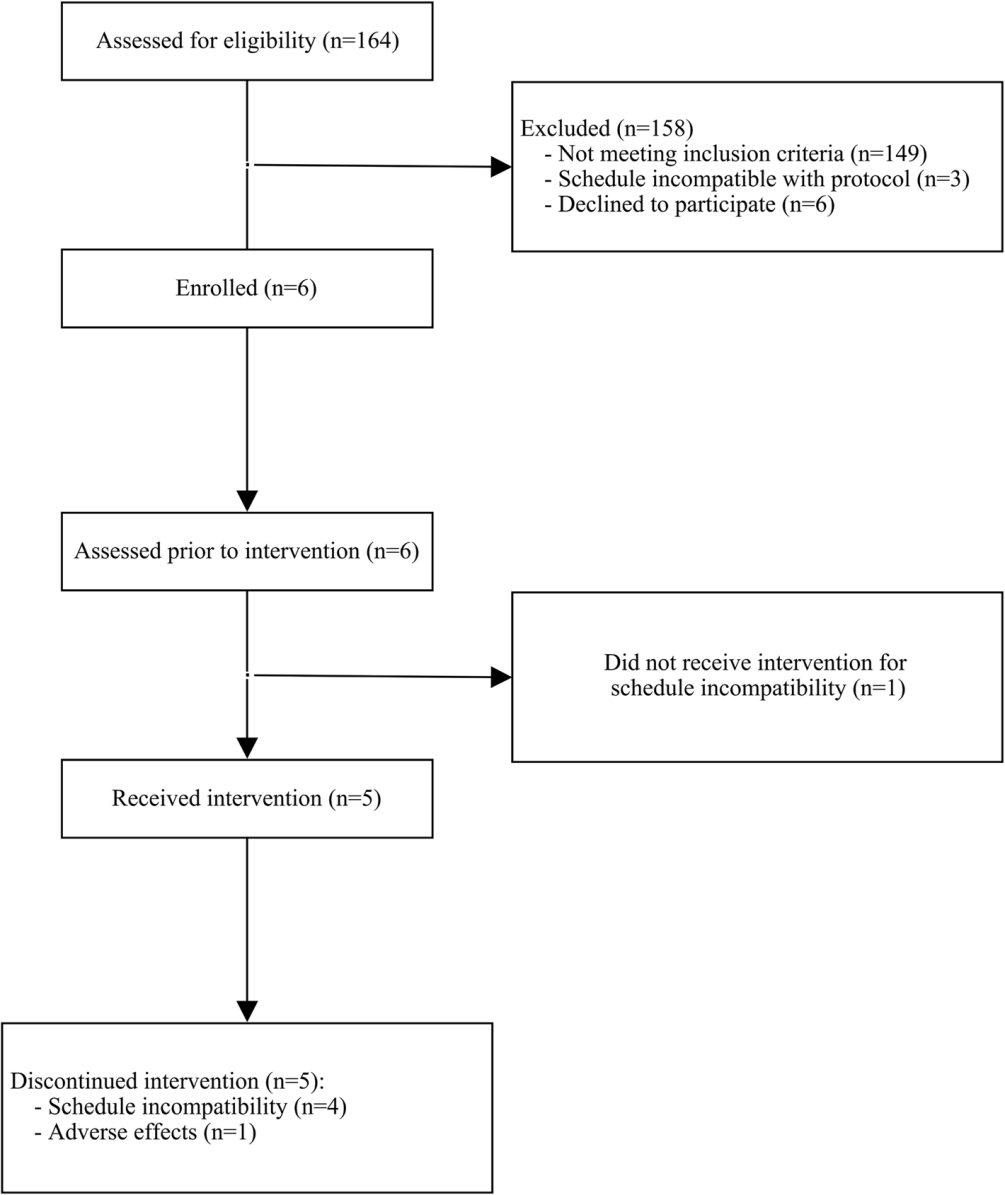
Study flow diagram

A total of 164 patients were admitted to the TBI unit at the IURDPM for intensive rehabilitation between September 2018 and January 2020; all were identified for this research project. From this group, 158 subjects were excluded (Fig. 1). The remaining six patients (five males and one female) with presumed attentional deficit related to TBI were recruited to receive the tDCS intervention (Fig. 2). Recruitment ended after we reached our arbitrary objective of five participants. Sociodemographic and clinical characteristics of participants are presented in Table 2.

**Table 2:** Patients’ characteristics

Patient	Sex/age (years)	Ethnic group	Education (years)	Time since TBI at T0 (weeks)	Severity/initial GCS	Cause of injury	Brain lesion (location)	Fracture	Surgery
1	M/70	White Canadian	16	11	Mod/15	Fall	SAH, SDH (R, L), contusion (RF, LF, LT, RT)	Nil	Nil
2	M/51	Black African	13	17.9	Mod/14	MVC	SDH (L)	Nil	Burr hole
3	F/74	White Canadian	9	6.4	MC/13	MVC	SAH, SDH (L), contusion (LF)	Nil	Nil
4	M/53	White Canadian	9	15.3	S/3	Fall	Contusion (LF, LT, O), DAI	Face	Nil
5	M/49	White Canadian	15	9.6	S/3	Fall	SAH, SDH (R), contusion (RF, RT)	Face, LP, LT	Nil
6	M/53	White Canadian	12	9.9	S/6	Assault	SAH (LF), SDH (L), EDH (RT)	Face, RT	Craniectomy
m±SD	58.3±10.8	N/A	12.3±2.9	11.7±4.2	N/A	N/A	N/A	N/A	N/A

*Abbreviations*: *m* mean, *SD* standard deviation, *M* male, *F* female, *MC* mild complicated, *Mod* moderate, *S* severe, *MVC* motor vehicle collision, *SAH* subarachnoid hemorrhage, *SDH* subdural hemorrhage, *SAH* subarachnoid hemorrhage, *EDH* epidural hemorrhage, *R* right, *L* left, *F* frontal, *T* temporal, *P* parietal, *O* occipital, *DAI* diffuse axonal injury

Participants received an average of 4.2 ± 3.4 stimulation sessions, representing 46.3% of the target number of interventions. None of the subjects went through the full planned nine-session protocol. Two subjects completed eight sessions, while one did not undergo any because of scheduling incompatibility. It took an average of 3.5 ± 1.3 weeks between admission and the first assessment, 3.1 ± 1.8 weeks between the first and the last assessment, for a total of 7.1 ± 2.8 weeks between the admission and the last evaluation, as opposed to the 4-week initial design plan.

Three patients completed the satisfaction questionnaire; all felt overall somewhat or very satisfied with their participation and would possibly or certainly recommend the project to someone else (Table 3).

**Table 3:** Satisfaction

Patients	Number of completed tDCS sessions	Satisfaction with information	Satisfaction with organization	Conflict with rehabilitation schedule	Course of the stimulation sessions	Course assessment sessions	Overall satisfaction	Recommend to someone else
1	8	Somewhat satisfied	Somewhat satisfied	Never	Somewhat satisfied	Somewhat satisfied	Somewhat satisfied	Possibly
2	2	-	-	-	-	-	-	-
3	0	-	-	-	-	-	-	-
4	8	Very satisfied	Very satisfied	Never	Very satisfied	Very satisfied	Very satisfied	Certainly
5	5	Very satisfied	Very satisfied	Never	Very satisfied	Very satisfied	Very satisfied	Certainly
6	2	-	-	-	-	-	-	-

Throughout the 25 completed tDCS sessions, 4 participants reported tingling, 3 skin redness, and 1 burning sensation. The effects were minor and temporary. One patient withdrew from the study due to intense headaches and fatigue following the stimulation and attributed it to tDCS (Table 4).

**Table 4:** Adverse effects

Adverse effects/patients	1	2	3	4	5	6	Total effects reported
Tingling	x			x	x	x	4
Skin redness	x				x	x	3
Burning sensation		x					1
Headache						x	1
Fatigue						x	1
Itching							0
Scalp pain							0
Neck pain							0
Sleepiness							0
Trouble concentrating							0
Acute mood change							0

All six patients completed the initial neuropsychological assessment (T0). Four patients completed neuropsychological assessments after stimulation. Despite prior clinical evaluation by physicians specialized in TBI, specific attention deficits were absent in four of the six subjects formally evaluated. In one patient, due to early hospital discharge, the final assessment was done at 1 week and after two neurostimulation sessions (T2). The remaining subject completed eight tDCS sessions and had attention impairment objectified, but no clinically significant change was seen at T8.

## Discussion

Satisfaction did not seem to be an obstacle to our intervention. The high rate of attrition before completion of nine sessions testifies to the complexity of organization of three sessions per week for 3 weeks with a simultaneous complete inpatient rehabilitation schedule. None of the participants received the intended nine intervention sessions and, in all subjects, stimulation sessions took longer than the originally scheduled 4 weeks. On this basis, we propose to reduce the number of weekly stimulation sessions.

Despite prior clinical evaluation by physicians specialized in TBI, specific attentional deficits were absent in four subjects as per the first neuropsychological evaluation. In retrospect, the inclusion should begin after the first neuropsychology evaluation. Considering the small size of our final sample, neuropsychological data were not used for a formal analysis but for feasibility assessment. With this in mind, the feasibility and tolerability of neuropsychological testing are adequate.

Challenges relating to subjects’ recruitment were the reluctant interest for neurostimulation which seems invasive and time consuming. An important finding is the exclusion of many potential participants. The exclusion criteria were determined according to previous studies. Published contraindications of tDCS are very restrictive, and as such, many patients did not meet the inclusion criteria (67% of subjects). Neurologic and psychiatric comorbidities, epilepsy, and substance abuse are common comorbidities in patients with TBI and hence restrict study inclusion.

The absence of a dedicated coordinator influenced adherence to the study protocol and communication between subjects, clinicians, and the research team. Second, our intensive functional rehabilitation setting is characterized by a short length of stay, medically active patients, and a relatively busy rehab schedule hindering participation. Moreover, because of memory impairment and dysexecutive functioning, it is challenging for patients with TBI to follow a schedule, and in order to promote social reintegration, patients are encouraged to leave their room during free time. Therefore, it was often difficult for the research team to schedule subjects, lengthening the recruitment and stimulation design. In addition, no designated neuropsychologist and no neuropsychology evaluation schedules were implemented, which also contributed to the delay between admission and first evaluation. When the neuropsychologist had both the clinical and research role, an ethical conflict was palpable in treating the patient and maintaining their objectivity in administering the chosen design tests. A single external neuropsychologist could ensure a homogenous administration and analysis of tests.

This feasibility study sheds light on the logistic and technical implication of neurostimulation. First, it allows professionals to discover this new interventional technique, its novel use on the TBI unit, and its promising potential. A series of recommendations are suggested in order to facilitate the implementation of similar projects in the future:

1. Assign an experienced research coordinator to improve recruitment, adherence to the protocol, and communication between subjects, clinicians, and the research team.
2. Assign an external neuropsychologist to avoid ethical conflict and to allow for efficient testing, scheduling, and analysis
3. Consider evaluation by a single neuropsychologist to ensure homogeneity of results
4. Consider stimulation sessions by a single researcher to ensure homogeneity of the intervention and data collection
5. Enroll subjects after the first neuropsychological assessment to improve screening for the cognitive deficit studied (i.e., attentional deficit)
6. Complete the study in an outpatient setting if discharge occurs before protocol ends
7. Plan no more than two NIBS sessions per week to realistically integrate them in a rehab schedule

## Conclusions

We believe that NIBS is a promising method for the treatment of attention deficits in patients with TBI undergoing inpatient rehabilitation. Based on this study and the existing literature, NIBS has its hurdles. Implementation of this newer technique comes with some challenges. Nevertheless, our study could stand as an aide for researchers to investigate the efficacy of neurostimulation in patients with TBI.

## Data Availability

The protocol and datasets generated during and/or analyzed during the current study are available from the corresponding author on reasonable request.

## Abbreviations

TBI: Traumatic brain injury
NIBS: Non-invasive brain neurostimulation
LTP: Long-term potentiation
LTD: Long-term depression
tDCS: Transcranial direct current stimulation
rTMS: Repetitive transcranial magnetic stimulation
DLPFC: Dorsolateral prefrontal cortex
TEA: Test of Everyday Attention
CPT-3: Conners’ Continuous Performance Test 3rd Edition
D-KEFS: Delis-Kaplan Executive Function System
WAIS-IV: Wechsler Adult Intelligence Scale–fourth edition
